# Intra-Articular Injections of Allogeneic Mesenchymal Stromal Cells vs. High Molecular Weight Hyaluronic Acid in Dogs With Osteoarthritis: Exploratory Data From a Double-Blind, Randomized, Prospective Clinical Trial

**DOI:** 10.3389/fvets.2022.890704

**Published:** 2022-06-07

**Authors:** Sohyun Kim, Lindsay Elam, Valerie Johnson, Ann Hess, Tracy Webb, Steven Dow, Felix Duerr

**Affiliations:** ^1^Department of Clinical Sciences, Colorado State University College of Veterinary Medicine and Biomedical Sciences, Fort Collins, CO, United States; ^2^Department of Statistics, Colorado State University, Fort Collins, CO, United States

**Keywords:** osteoarthritis, dog, stem cell therapeutics, hyaluronic acid, regenerative medicine

## Abstract

This double-blind, randomized, prospective clinical trial was conducted to obtain exploratory data comparing the efficacy of intra-articular allogeneic mesenchymal stem/stromal cells (MSC) to high molecular weight hyaluronic acid (HA) for the treatment of pain associated with canine osteoarthritis (OA). Objective gait analysis (%Body Weight Distribution, %BWD), accelerometry, clinical metrology instruments and veterinary exams were used as outcome measures during various time points throughout the 48-week study period. Fourteen dogs with elbow or coxofemoral OA were enrolled and assigned in a 2:1 ratio to the treatment groups. Each patient received a set of two injections 4 weeks apart. Self-limiting joint flare was observed in seven patients, with six of these in the MSC group. Ten patients completed all follow-up appointments. Both treatment groups showed evidence of mild improvement following the treatment, but the results were inconsistent among the various outcome measures assessed. Overall, dogs enrolled in the HA group showed greater improvement compared to the MSC group. The primary outcome measure, %BWD, showed evidence of improvement, when compared to baseline values, at 36 weeks after injection for the HA group only (*p* = 0.048, estimated difference: 4.7). Similarly, when treatment groups were compared, evidence of a difference between treatment groups (with the HA-group showing greater improvement) were identified for weeks 24 and 36 (*p* = 0.02 and 0.01, respectively). The small sample size of this exploratory study does not allow firm conclusions. However, until studies with larger sample sizes are available, the current literature combined with our data do not support the clinical use of intra-articular MSC therapy over high molecular weight HA for the treatment of canine OA at this time.

## Introduction

Osteoarthritis (OA) is the most common human joint disorder in the world, estimated to clinically impact ~30 million adults in the US (1). Based on an unpublished survey of 200 veterinarians performed in 1996, it is frequently stated that at least 20% of dogs over 1 year of age are affected (2). In a recent publication, Wright et al. reported an even higher prevalence of 38% in a study population of 500 dogs. These dogs were not previously diagnosed with OA, did not receive medications for treatment of OA, and presented for routine care (3). On the other hand, review of a large veterinary database from primary care facilities in the UK described the overall 1-year period prevalence of OA to be 2.3–2.5%, with certain breeds showing a higher prevalence (e.g., Golden Retriever, 7.4%; Labrador, 6.1%) (4, 5). However, the latter numbers likely heavily underestimate the true prevalence since they are based on retrospective medical record data review only (6).

Regardless of the true prevalence of OA, because of its progressive and debilitating nature, OA poses a significant welfare issue to canines and humans alike. In a recent epidemiologic study investigating more than 12,000 German Shepherd Dogs in the UK, osteoarthritis/musculoskeletal disease was the most common cause of death; surpassing even neoplasia (7). Yet, there still is a lack of treatment options that consistently offer pain relief and improve quality of life without the risk of substantial adverse effects. Currently, there are multiple treatment options to address the pain associated with OA ranging from surgery (e.g., arthroscopy, joint replacement) to a myriad of medical management interventions (e.g., weight loss, anti-inflammatories, analgesics, nutritional supplements, physical rehabilitation, acupuncture, shockwave therapy, etc.). A multi-modal approach is often pursued to enhance treatment efficacy while attempting to minimize systemic adverse effects (8). Targeted local therapy, such as intra-articular injections, has been a developing area of interest. Several intra-articular treatments have been reported in veterinary medicine, the most common being corticosteroids, hyaluronic acid (HA), platelet-rich plasma (PRP), and mesenchymal stem/stromal cells (MSC) (9, 10).

Viscosupplementation of joints has been used for decades in animal and humans (11). Hyaluronic acid, a naturally occurring non-sulfated glycosaminoglycan with excellent viscoelastic properties, is crucial for normal joint function. Because of its ability to trap water, it aids in providing compressive strength to articular cartilage, thereby acting as a natural shock absorber. The benefits of intra-articular injection of HA include anti-inflammatory, analgesic, and chondroprotective effects. HA can be produced either by extraction from animal tissues (e.g., chicken combs) or *in vitro* by bacterial fermentation. Independent of the production method, it can be stored at room temperature and is readily available off-the-shelf (12). Many studies have shown that HA can be beneficial in patients with OA, however, the magnitude of improvement is generally accepted to be small and may depend upon the molecular weight of the product (11–14).

Regenerative medicine has recently gained popularity in the treatment of OA. Intra-articular therapy with MSCs is purported to alleviate OA pain *via* several pathways (15). Intra-articular MSCs are theorized to stimulate a release of chemical mediators that improve the secretion of growth factors, which enhance cartilage repair and regeneration through processes such as cell migration, proliferation, differentiation, and matrix synthesis, though the exact mechanism remains to be elucidated (16). Additionally, MSCs have immunomodulatory properties that can attenuate the immune responses in the host by inhibiting activation of T and B lymphocytes and natural killer cells, which are known to play a role in the development and progression of OA (17).

MSC are obtained from various sources (e.g., bone marrow, placenta, umbilical cord, etc.), but adipose-derived MSC have the particular advantage of being both abundant and a more easily harvested resource (18). Both autologous and allogeneic adipose-derived MSC have been used in veterinary medicine, though to date, there is no consensus on which is safer and more effective (19). Treatment with autologous adipose-derived MSC decreases risk of infectious disease transfer and immunogenicity issues but has some inherent morbidity associated with harvesting tissue; studies in mice have found that the number and quality of cells decrease with age of the donor (20), which may limit application in patients suffering from OA as many are in the middle to older age group. While there are some disadvantages to the use of allogeneic adipose-derived MSC such as potential infectious disease transfer and overexpansion (to attain large stocks of cells), their use has the benefit of using healthy, young donors to maximize cell quantity and quality, and eliminating any morbidity associated with the harvesting procedure for the patient that is receiving the MSC treatment (21).

Several studies have evaluated the efficacy of MSC for treatment of canine OA with promising results in the last decade (9, 22). Nevertheless, studies have not confirmed whether the effects of MSC are superior and/or safer than other existing intra-articular treatment options (23, 24). To date, there is a lack of conclusive data comparing intra-articular MSC treatment with readily available, off-the-shelf treatments such as hyaluronic acid (HA). The purpose of this study was to collect exploratory data comparing the efficacy of intra-articular allogeneic MSC (Allo-MSC) to HA for the treatment of pain associated with canine OA. We hypothesized that the Allo-MSC treatment group would demonstrate improved outcome (based on the primary outcome measure, %Body Weight Distribution [%BWD]) compared to the HA treatment group.

## Materials and Methods

### Patient Selection and Study Protocol

The ARRIVE 2.0 guidelines [Reporting of Animal Research: Reporting of *in vivo* Experiments (25)] were followed in designing and reporting of this research. The trial was a double-blind, randomized, prospective clinical study which recruited dogs with lameness attributable to naturally occurring OA of the coxofemoral or elbow joint. The study was approved by the institutional review board (Clinical Review Board #2017-129), and owner consent was obtained for each case. Given the exploratory nature of this study, no sample size calculation was performed. Client owned dogs presented to Colorado State University were evaluated by a board-certified orthopedic surgeon and overseeing trial veterinarian at the enrollment visit. Inclusion criteria were defined as follows: body weight over 10 kilograms, radiographic evidence of OA of the joint to be treated, and Canine Brief Pain Inventory (CBPI) values for pain severity score (PSS) and pain interference score (PIS) of ≥2 for each in their initial owner questionnaire. The patients were required to display a visually identifiable lameness on subjective gait analysis. Additionally, objective gait analysis was performed, and %BWD had to be outside of a previously reported reference range for the affected limb (26). Patients had to have consistent clinical signs that had been present for at least 4 weeks prior to enrollment. There were no age or breed restrictions. Only patients with a score of ≥3 based on a previously described (27) Subjective Orthopedic Scoring system (SOS) grading for combined scores of “Lameness at walk” and “Lameness at trot” were included. Lameness secondary to OA could be bilateral, but one side had to be worse based on both subjective (SOS grading) and objective (%BWD) measurements. Exclusion criteria included concurrent systemic diseases (e.g., Cushing's disease, diabetes mellitus, chronic liver, or kidney disease), patients unable to safely undergo sedation (e.g., cardiomyopathy), inconsistent OA management over the prior 4 weeks, and owners that were unable to follow the proposed recheck schedule and/or complete questionnaires as outlined in the study protocol over the 1-year study duration.

### Outcome Measures

**Figure 1:**
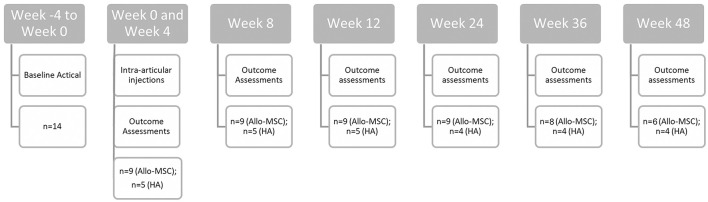
Graphical representation of the study timeline and sample size.

Various outcome measures were scheduled to be collected at weeks 0, 4, 8, 12, 24, 36, and 48 of the trial (see Figure 1). Objective gait analysis was performed using a pressure sensitive walkway (Tekscan HR Walkway^™^ 6 VersaTek System), and %BWD, defined as {[PVF (N) of the limb/total PVF (N) of all four limbs in one gait cycle] × 100}, was collected as previously described (27). Briefly, animals were evaluated at the walk or trot (based on their preference), but the velocity was kept consistent between time points. For data analysis, trials from an individual dog were only considered for analysis if they fell within a velocity range ≤0.3 m/s between time points. The goal was to obtain trials with a consistent velocity, in a straight line, without lateralization of the head, pulling on the lead, or stepping off the PSW (three in each direction). The protocol was adjusted as needed with the goal of capturing at least one valid trial in either direction. If animals only tolerated walking in one direction, then only trials in that direction were acquired (i.e., aiming for six valid trials in one direction). The body weight and the number of trials required were recorded at each time point.

Accelerometers were used to objectively measure physical activity data. The patients had the accelerometer collars placed for minimum of 4 weeks before the first injection to obtain baseline activity level (i.e., week −4 to week 0). Total activity counts (AC) and activity intensity was collected as previously described using the Actical accelerometer (27, 28). Briefly, the epoch was set to 60 s, and only AC data with a minimum of 140 min of recorded activity per day was used for analysis. Data was recorded as the automatically generated number of minutes per week spent in the different activity categories assigned by the Actical device (28). Data was recorded continuously throughout the entire study. Accelerometry data was pre-processed to average the data over 4 weeks including the time point of interest (0, 12, 24, and 36 weeks), and analysis was performed the same as was performed on the raw data. Activity data was only analyzed for those time points where sufficient data was available.

Owner outcome assessment of pain and mobility at home was performed using validated questionnaires (CBPI and Client Specific Outcome Measures, CSOM). CSOM questions based on previously published information were divided into activity and behavior categories and scoring was performed as previously described (21). Briefly, the owners were asked to pick 5 time and place specific activities and grade them on a 1–5 scale (1 = no problem, 2 = a little problematic, 3 = quite problematic, 4 = severely problematic, and 5 = impossible) and pick 3 activities related to behavior, also graded on a 1–5 scale (1 = significantly less than normal, 2 = less than normal, 3 = normal amount, 4 = more than normal, and 5 = significantly more than normal). These questions were normalized for analysis with higher numbers indicating worsening of symptoms and lower numbers indicating improvement of symptoms. The CBPI questionnaire was used in unedited form as recommended by the developer of the questionnaire (29). The same owner was required to complete all questionnaires at each time point in a dependent interview process either in-person or over the phone due to pandemic-related restrictions of in-person visits.

### Allo-MSC Preparation

MSCs were generated from adipose tissues collected from the inguinal region and/or abdomen of anesthetized, purpose-bred research hound dogs <4 years of age used in a veterinary teaching laboratory. Prior to use of adipose tissue samples dogs were tested for infectious diseases and routine laboratory testing was performed as previously described (30). The adipose tissues were collagenase-digested (collagenase 1 mg/ml Sigma-Aldrich St. Louis MO) for 30 min at 37°C then centrifuged at 1,050× g for 5 min, triturated, and then recentrifuged. The resulting stromal vascular fraction was plated for enrichment and expansion of MSC in low glucose Dulbecco's Modified Eagle medium with 5% essential and non-essential amino acids, glutamine and penicillin-streptomycin (Gibco, Thermofisher Scientific Waltham MA), and 15% heat-inactivated fetal bovine serum (VWR, Radnor PA). Cells were incubated at 37°C in 5% CO_2_ and passaged when 80–95% confluent, harvested on the day of injection by detaching cells using 1% trypsin (Sigma-Aldrich, St. Louis MO), washed three times with Dulbeccos Phosphate Buffered Saline (DPBS;Sigma-Aldrich, St. Louis MO), and resuspended in 2 ml DPBS for injection. MSCs were used between passages 2–5, and cell count and viability assessments were performed by manual count using a hemocytometer and trypan blue dye to detect dead cells. Cell viability was required to be >95%, and the phenotype, morphology, and trilineage differentiation capacity of the MSCs was required to be consistent with that previously described for canine MSC (30). Prior to initiation of the study, cell lines to be utilized in this study were assessed for phenotypic markers associated with canine MSC (CD105, CD73, CD44, CD45, CD34) *via* flow cytometry. They were additionally examined for the ability to differentiate into chondrocytes, adipocytes, and osteocytes utilizing StemPro chondrogenesis, osteogenesis, and adipogenesis kits per manufacturer's instructions (Gibco, Thermofisher Scientific, Waltham MA). Prior to MSC injection an aliquot of cells was also aseptically collected and plated to verify that the cells were free of bacterial, mycoplasma or fungal contamination. Briefly, the cell suspension was cultured on sheep blood agar, MacConkey agar (BD, Franklin Lake NJ), mycoplasma agar (Udder Health, Bellingham WA) and Sabaroud Dextrose agar (BD, Franklin Lake NJ). Fungal cultures were incubated at room temperature for 30 days, mycoplasma cultures were incubated at 37°C with 5% CO_2_ for 7 days, and blood agar and MacConkey plates were cultured at 37°C for 48 h after which cultures were considered negative. Prior to injection of the last two dogs with Allo-MSC, the cell culture protocol was altered to incubate the cells in serum free media for 48 h prior to injection using StemPro xeno free media (SFM; Gibco, Thermofisher Scientific, Waltham MA) because of the joint flare observed in two patients.

### Intra-Articular Injection Administration

Dogs were randomly assigned to the Allo-MSC or HA group, in a 2:1 ratio, respectively. All clinicians involved in collecting outcome measurement data were blinded to the treatment administered by covering the injectate syringe with parafilm (and transferring the HA to a regular syringe). All dogs received intra-articular injections of either Allo-MSC (10 × 10^6^ MSCs) or HA (SYNVISC-ONE, Genzyme, Ridgefield, New Jersey; produced from chicken combs with an average molecular weight 6,000,000 daltons for hylan A; 2 mls per joint for dogs >15 kg and 1 ml per joint for dogs <15 kg; 4.8 mg/ml,) at 2 time points 4 weeks apart (i.e., week 0 and 4). For administration of intra-articular injections, patients were sedated with Dexmedetomidine (5 mcg/kg IV) and Hydromorphone (0.05 mg/kg IV) and reversed with Atipamezole (50 mcg/kg IM) following the procedure. Vital parameters were monitored throughout sedation. The affected joint was clipped and prepped using standard aseptic technique. The elbow joint was identified using palpation of local landmarks to guide the injection; coxofemoral joints were injected using ultrasound guidance. To confirm injection into the joint, aspiration of joint fluid prior to administration (for elbow joints) or verification of distension of the joint capsule *via* ultrasound (for coxofemoral joints) was performed. For cases with bilateral lameness, clinical judgment was used to determine if injection of both joints was indicated (i.e., patients with bilateral lameness were allowed to receive treatment, with either HA or Allo-MSC, in both joints). Joint flare was defined as worsening of lameness within 48 h after intra-articular injection. To identify post-injection joint flare, owners were called ~48 h after the injection to inquire whether their dog's lameness had worsened (i.e., joint flare), stayed the same or improved. If worsening of lameness persisted beyond a few days, owners were asked to return the patient for evaluation.

### Statistical Analysis

Statistical analysis was completed using a commercially available software package (SAS 9.4 software, SAS Institute Inc., Cary, NC, USA). Summary statistics (mean, standard deviation, min, median, max) were calculated for each variable, treatment, and time point. Residual diagnostic plots were used to evaluate model assumptions of normality and equal variance. A mixed model was fit for each response variable separately. Specifically, treatment and time and treatment by time interaction were included as fixed effects. To account for repeated measures across time, dog was included in the model as a random effect. For each time point, comparisons were made between treatments. For each treatment, comparisons between downstream time points vs. baseline (Week 0) were performed using Dunnett's method. For Actical data specifically, 4 weeks of data preceding the time point of interest (i.e., weeks −4 to week 0 for week 0/baseline; weeks 8–12 for week 12; weeks 20–24 for week 24, and weeks 32–36 for week 36) were averaged and compared to baseline, using Dunnett's method as previously described. If a dog had a sedentary value <5,000, this entire week of data was omitted prior to averaging. If a dog had fewer than 3 weeks of observations contributing to the average, this observation was excluded. Actical “Vigorous” data was not used for formal analysis because most values were zero.

## Results

A total of 14 dogs were enrolled, consisting of 6 male castrated, 2 male intact, 5 female spayed, and 1 female intact dogs (Table 1). The mean age was 8.75 years (range 1.5–13 years); the mean body weight was 30.1 kg (range 11–45 kg). There were eight patients with elbow OA and six patients with coxofemoral OA. From the initial population of 14 patients enrolled, 10 patients (Allo-MSC:*n* = 6; HA:*n* = 4) completed all follow-up appointments over the 48-week period. Two patients were euthanized prior to completion for reasons unrelated to the study (at week 36 and 48, respectively). Another patient (in the HA group) was withdrawn at 12 weeks as the owner elected to go forward with a bilateral femoral head and neck ostectomy, and one was lost to follow-up for their final study visit.

**Table 1:** Overview of the study participants.

**Breed**	**Group**	**Sex**	**Age (years)**	**Weight (kg)**	**Site of injection**	**Study endpoint**
Entlebucher mountain dog	ALLO-MSC	MC	10	25.3	Right elbow	Euthanized
Mixed breed dog	HA	FS	13	23.3	Right elbow	Completed
Chesapeake bay retriever	ALLO-MSC	FS	8	27.7	Left hip	Completed
Labrador Retriever	ALLO-MSC	FS	10	40.5	Both hips	Completed
Labrador retriever	HA	MC	8	31.6	Right elbow	Completed
Border collie mix	HA	FS	6	18.1	Both hips	Surgical treatment pursued
German shepherd dog	ALLO-MSC	MC	10	45	Right elbow	Completed
Siberian husky	ALLO-MSC	FI	3	21.4	Right hip	Completed
Golden retriever	ALLO-MSC	MC	12	34	Left elbow	Euthanized
German shepherd dog	HA	MI	1.5	39.4	Left elbow	Completed
Labrador retriever	ALLO-MSC	MC	10	37	Left elbow	Completed
Labrador retriever	ALLO-MSC	MI	12	33.5	Left hip	Completed
Labrador retriever	ALLO-MSC	FS	8	34.3	Right hip	Lost to follow-up
West highland terrier	HA	MC	11	11	Right elbow	Completed

*MC, male castrated; FS, female spayed*.

The most commonly reported adverse event after intra-articular injection was self-limiting joint flare after the injection (*n* = 7, with 6/7 from the Allo-MSC treatment group; *n* = 3 elbow joints and *n* = 3 coxofemoral joints in the Allo-MSC treatment group and *n* = 1 elbow in the HA treatment group) that resolved either without treatment or with short-term anti-inflammatory medications and ice-packing. Two patients (one patient in the Allo-MSC treatment group and one patient in the HA treatment group) were noted to have self-limiting joint flare for the first 2 days following the injection, and then presented again 2–3 weeks later for acute worsening of lameness (toe-touching lameness of the affected limb without inciting cause). Aerobic and anaerobic synovial fluid culture and cytology performed for both patients did not show evidence of joint infection, however, antibiotic therapy (cephalexin for the patient in the Allo-MSC treatment group and amoxicillin trihydrate/clavulanate potassium for the patient in the HA treatment group) was instituted regardless. In both cases, culture and cytology was repeated after 6–8 weeks of antibiotic therapy. Both cases were maintained in the study for continued follow-up due to clinical improvement, and the lack of positive synovial fluid cultures and cytologic evidence of septic arthritis (i.e., inability to attribute the flare to iatrogenic septic arthritis).

**Figure 2:**
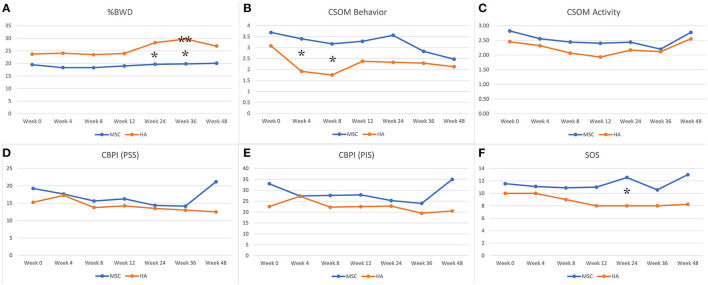
Graphical representation of data for both groups for %Body Weight Distribution (%BWD), Client Specific Outcome Measures (CSOM), Canine Brief Pain Inventory (CBPI) (PSS = pain severity score and PIS = pain interference score), and Subjective Orthopedic Scoring system (SOS). *Indicates evidence of a difference for comparison between the mean values for the two treatment groups. **Indicates evidence of a difference for comparison of mean baseline value to the subsequent time point within the treatment group. **(A)** %BWD, **(B)** CSOM behavior, **(C)** CSOM activity, **(D)** CBPI (PSS), **(E)** CBPI (PIS), and **(F)** SOS.

Overall, both treatment groups showed evidence of mild improvement following the treatment, but the results were inconsistent among outcome measures assessed (see Appendix for details of all outcome measures and Figure 2). The primary outcome measure, %BWD of the most affected limb, showed evidence of improvement in the HA group when compared to baseline at 36 weeks (*p* = 0.048), while the Allo-MSC group did not exhibit any major degree of improvement when compared to baseline at subsequent post-treatment time point (Table 2). When treatment groups were compared, evidence of a difference between treatment groups were identified for weeks 24 and 36 (*p* = 0.02 and 0.01 respectively), with the HA group showing greater improvement.

**Table 2:** Comparison between the mean values (±SD) of the primary outcome measure (%BWD of the affected limb) for the two treatment groups at each time point.

**Group**	**Week 0**	**Week 4**	**Week 8**	**Week 12**	**Week 24**	**Week 36**	**Week 48**
ALLO-MSC	19.48 (4.58)	18.33 (3.99)	18.3 (4.89)	19 (5.42)	19.67 (4.79)^a^	19.84 (6.84)^b^	20.07 (6.17)
*n=*	*9*	*9*	*9*	*9*	*9*	*8*	*6*
HA	23.72 (6.27)	24.05 (5.98)	23.5 (6.07)	23.96 (4.48)	28.25 (4.12)^a^	29.73 (4.58)^b^^*^	26.9 (4.36)
*n=*	*5*	*5*	*5*	*5*	*4*	*4*	*4*

a,b *Values with the same superscript indicate evidence of difference (p < 0.05) between the mean values for the two treatment groups at the respective time point.*

* *Values with an asterisk indicate evidence of difference (p < 0.05) for the comparison of mean baseline values to the respective time point within the treatment group.*

The accelerometry data revealed consistently higher total activity levels in the Allo-MSC group (including baseline), but no difference was noted when compared to total activity levels in the HA group. Evidence of a decrease in the immediate post-injection treatment series time point (12 weeks) within the Allo-MSC group was noted compared to baseline with a decrease in the light (*p* = 0.02) and moderate (*p* = 0.03) activity counts and an increase in sedentary activity counts (*p* = 0.01).

Despite both groups showing a decrease in CSOM behavior questionnaires after completion of the treatment series, there was disparity noted between the two groups (*p* = 0.04), with the HA group showing a greater degree of improvement. Similarly, for comparisons between treatment groups, a difference between groups was evident at the 24-week time point for comparison of SOS (*p* = 0.05; with the HA group showing greater improvement). No evidence of difference between treatment groups or between time points within each group were identified for any other outcome measures.

## Discussion

Several recent studies have evaluated intra-articular injections with MSC in dogs with OA, many suggesting that there may be some benefit associated with this treatment (9, 22–24). However, most of these studies either lack appropriate outcome measures, lack a control group, or the control group consists of no treatment. While choosing no treatment as the control obviously increases the ability to detect differences between groups, comparison to HA is perhaps more clinically relevant. Given that the mechanism of action of HA is well-defined and the treatment is simple, safe, and comparably inexpensive, it serves as a logical clinical alternative comparison group. This is the first canine study comparing the clinical efficacy of repeated intra-articular injections of HA to Allo-MSC. While we expected improvement in the HA treatment group, we did not expect it to have comparable, if not better, treatment efficacy compared to the Allo-MSC treatment group.

Our study results revealed overall mild improvement following the two treatment injections in both groups but did not reveal one treatment to be superior to the other across multiple outcome measures. Owner questionnaires and SOS results were mildly improved in both groups with the HA group showing a greater degree of improvement in the CSOM questionnaire at week 12 and with SOS at week 24. It is important to note that both of these outcome measures are subject to the “caregiver placebo” effect (31). Particularly because there was no placebo arm, the caregivers (veterinarians for SOS and owners for CBPI and CSOM scoring) are likely to report a benefit for both treatments. As such, objective outcome measures are most relevant for this particular study design when it comes to determining overall efficacy of the treatment rather than comparison of the two treatments. For objective outcomes measures, the HA group demonstrated greater improvement in %BWD (an outcome measure unaffected by caregiver placebo effect) compared to the Allo-MSC group at the 24 and 36 week time points. The accelerometry data showed no evidence of sustained increase in activity post treatment for either group.

There are many reasons that may explain the findings of the present study, including the potential superiority of the high molecular weight (HMW) HA used, potential inferiority of the Allo-MSC used (compared to MSC types used in previous studies), the study design, and/or the utilized outcome measures. For example, while accelerometers have been evaluated in several research studies, there are some concerns regarding the ability of this data to identify differences in activity patterns due to the number of variables [e.g., owner-induced activity, data collection and processing, accuracy of the devices, and averaging of data resulting in the inability to detect short-term changes such as changes in sleep and activity patterns (32)]. While OGA is a well-accepted outcome measure, and %BWD has been described as the most accurate outcome measure when using PSW in a heterogenous study population (33), there many factors that influence OGA data (34). Furthermore, it only captures single, brief time points, which is why a set of diverse outcome measures should be considered in clinical trials (28).

Previous studies using HA in humans and canines with OA reported several beneficial effects including anti-inflammatory, analgesic, and chondroprotective properties (11, 12). We chose to use HA as a comparison group because HA is readily available to clinicians when considering intra-articular injections. Alternatively, we could have chosen PRP or saline for our control group. We did not opt for PRP because there is still substantial controversy regarding the ideal treatment regime and constitution of PRP, making it difficult to compare studies using different types of PRP. We did not choose saline injections because it is not a clinically applied treatment and therefore a superiority of MSC over saline would not be as clinically relevant. The molecular weight of native HA has been reported to be ~4,000–10,000 kilo Daltons (kDa) in humans, and 2,000–3,000 kDa in horses (12). While there is no clear definition of high vs. low molecular weight, products with a molecular weight of <1,500 kDa are frequently considered LMW while products with a molecular weight >5,000 kDa are frequently considered HMW. We chose HMW HA for this study based on several studies suggesting a superiority to LMW HA (12). There has been some controversy over the efficacy of HA for the treatment of knee OA in humans, with some authorities suggesting that treatment does not result in a clinically relevant difference. A recent study, however, found that these results may be due to the consolidation of different molecular weights of HA in meta-analyses with HMW HA resulting in a clinically relevant benefit (14). Cook et al. compared the efficacy and safety of LMW and cross-linked HMW HA intra-articular injections in surgically induced stifle OA in dogs, using saline injections as a control. Their findings suggested that overall, treatment with HA showed more improvements in pain, function, and range of motion compared to the saline control, but the HMW HA treatment group demonstrated the most improvements (35). Alves et al. described reduction in pain and functional improvements when evaluating the effect of a single intra-articular injection of HMW HA in canines with naturally occurring hip OA (36). Our preliminary results are in line with these previous reports regarding efficacy of HMW HA.

Unfortunately, MSC treatment is not standardized, and many variables exist that may have substantial impact on the outcome, including but not limited to the source and number of cells, culture expansion methods, media components, cryopreservation, and administration frequency. The present study utilized culture-expanded, adipose-derived Allo-MSC. It is possible that the cells used in the present study provide inferior benefits compared to MSC used in other studies. However, the cells used in this study displayed phenotypic and functional characteristics consistent with commonly accepted definitions of MSCs which were similar to descriptions in other published studies using canine Allo-MSC (9, 21, 37). Allogeneic cells have been reported to be safe and have several key advantages over autologous cells and have been used to safely treat canine patients with OA (9, 21, 37). Some of the most attractive advantages of Allo-MSC include the ability to expand and bank Allo-MSC, and the ability to establish cells lines that may produce more uniform cellular therapy to allow for more predictable response (19). Other advantages include the elimination of a separate cell collection procedure and the ability to source cells from younger, healthy donors. While there is potential to develop Allo-MSC as another “off-the-shelf” therapy that may benefit a larger scale of veterinary patients, further research into the safety and efficacy of the treatment must be pursued prior to the widespread commercialization efforts.

In this study, seven patients experienced some degree of joint flare, and six out of seven of those patients were in the Allo-MSC group. This is similar to findings reported in other studies involving intra-articular injections, particularly repeat injections of Allo-MSC (18, 38). Of the six joint flares in the Allo-MSC group, two were reported to have flare after both injections, two had flare only after the first injection, and another two had flare only after the second injection. Joswig et al. suggested that the xeno-contaminants used to produce Allo-MSC, such as Fetal Bovine Serum (FBS), may cause development of recipient antibodies to the foreign bovine proteins with subsequent rejection of cellular therapy in equine models. This may result in the joint flare observed in patients, typically reported after repeated intra-articular injections of Allo-MSC (39). In this scenario, one would expect that joint flare would be worse on repeated injections due to sensitization either to foreign MHC molecules or to sensitization against FBS proteins, unless the animal had been previously exposed. The reports of joint flares appear to be distributed inconsistently with this theory in our study, although previous exposure was not definitively ruled out. Further investigation comparing the various preparation methods, including the use of serum free media, as well as the cause of potential inefficacy and joint flare are required before appropriate clinical recommendations can be made.

One of the obvious limitations of the present study is inherent to the small sample size associated with the exploratory nature of this clinical trial. While there are many published studies with small sample sizes in veterinary medicine, it is important to understand their limitations. Specifically, the possibility of identifying a statistically significant difference that does not reflect a true effect, thereby producing misleading results (40). While the term “Pilot Study” is frequently used in veterinary medicine, Rishniw et al. (41) suggested that this term most frequently represents a “deficiency signal” to editors, indicating an underpowered study. We therefore describe the current study as an “Exploratory Study,” indicating that the purpose of this study is not to provide conclusive results, but rather to generate exploratory data that can be used to determine future hypotheses and study designs. Based on the results of the presented data, one future research hypothesis may be that both HA and Allo-MSC provide mild benefit for the treatment of canine OA, yet that there is no difference between the two products. Besides the small sample size, there are several other study weaknesses that should be considered for future research aiming to answer this potential hypothesis. Examples include the heterogenous study population, inclusion of animals with both elbow and hip OA, absence of biomarker evaluation, and lack of a control group. Additionally, due to missing data points, the sample sizes varied across response variables and time points. The inconsistency in between outcome measures illustrates that more robust studies are required. Therefore, our findings should not be over interpreted and be limited to guide future research rather than draw firm conclusions regarding the efficacy of the products tested. Larger controlled trials are clearly needed to confirm or deny the preliminary findings from the present study.

Overall, the current literature provides insufficient evidence to justify intra-articular MSC injections for the treatment of canine OA (24). Concerns have been raised that the popularization of MSC is driven by commercial interests rather than the pet's best interest (23). The data presented here further question the routine clinical use of intra-articular Allo-MSC at this time. The wide availability, off-the-shelf nature, safety, and possibly lower cost make HA a potential treatment standard to which novel products should be compared (and expected to be superior to) prior to widespread clinical use. Further studies defining and investigating the potential greater clinical benefit of HMW HA for the treatment of canine OA should be considered. Future studies may consider adding a true placebo arm, more advanced data analysis of the accelerometer data [e.g., functional linear modeling (32)], and/or more frequent OGA data collection.

## Data Availability Statement

The datasets presented in this study can be found in online repositories. The names of the repository/repositories and accession number(s) can be found in the article/Supplementary Material.

## Ethics Statement

The animal study was reviewed and approved by Clinical Review Board and Animal Care and Use Committee of Colorado State University. Written informed consent was obtained from the owners for the participation of their animals in this study.

## Author Contributions

SK, VJ, AH, TW, SD, and FD contributed to the conception and design of the study. SK organized the data and wrote the first draft of the manuscript. All authors contributed to manuscript revision, read, and approved the submitted version.

## Funding

This study was partially funded by the Shipley Family Foundation.

## Conflict of Interest

The authors declare that the research was conducted in the absence of any commercial or financial relationships that could be construed as a potential conflict of interest.

## Publisher's Note

All claims expressed in this article are solely those of the authors and do not necessarily represent those of their affiliated organizations, or those of the publisher, the editors and the reviewers. Any product that may be evaluated in this article, or claim that may be made by its manufacturer, is not guaranteed or endorsed by the publisher.
